# Herpes Simplex Virus Type 1 Penetrates the Basement Membrane in Human Nasal Respiratory Mucosa

**DOI:** 10.1371/journal.pone.0022160

**Published:** 2011-07-15

**Authors:** Sarah Glorieux, Claus Bachert, Herman W. Favoreel, Annelies P. Vandekerckhove, Lennert Steukers, Anamaria Rekecki, Wim Van den Broeck, Joline Goossens, Siska Croubels, Reginald F. Clayton, Hans J. Nauwynck

**Affiliations:** 1 Laboratory of Virology, Faculty of Veterinary Medicine, Ghent University, Merelbeke, Belgium; 2 Department of Otorhinolaryngology, University Hospital Ghent, Ghent, Belgium; 3 Laboratory of Immunology, Faculty of Veterinary Medicine, Ghent University, Merelbeke, Belgium; 4 Department of Morphology, Faculty of Veterinary Medicine, Ghent University, Merelbeke, Belgium; 5 Department of Pharmacology, Toxicology and Biochemistry, Faculty of Veterinary Medicine, Ghent University, Merelbeke, Belgium; 6 Tibotec BVBA, Beerse, Belgium; University of Minnesota, United States of America

## Abstract

**Background:**

Herpes simplex virus infections are highly prevalent in humans. However, the current therapeutics suffer important drawbacks such as limited results in neonates, increasing occurrence of resistance and impeded treatment of stromal infections. Remarkably, interactions of herpesviruses with human mucosa, the locus of infection, remain poorly understood and the underlying mechanisms in stromal infection remain controversial.

**Methodology/Principal Findings:**

A human model consisting of nasal respiratory mucosa explants was characterised. Viability and integrity were examined during 96 h of cultivation. HSV1-mucosa interactions were analysed. In particular, we investigated whether HSV1 is able to reach the stroma.

Explant viability and integrity remained preserved. HSV1 induced rounding up and loosening of epithelial cells with very few apoptotic and necrotic cells observed. Following 16–24 h of infection, HSV1 penetrated the basement membrane and replicated in the underlying lamina propria.

**Conclusions/Significance:**

This human explant model can be used to study virus-mucosa interactions and viral mucosal invasion mechanisms. Using this model, our results provide a novel insight into the HSV1 stromal invasion mechanism and for the first time directly demonstrate that HSV1 can penetrate the basement membrane.

## Introduction

Alphaherpesviruses constitute the largest subfamily of the *Herpesviridae* and consist of closely related pathogens of man and animals. The human alphaherpesviruses are comprised of the two antigenically distinct herpes simplex viruses type 1 (HSV1) and type 2 (HSV2) and varicella-zoster virus (VZV). HSV is a clinically important pathogen where HSV1 generally causes oral blisters and HSV2 causes genital ulcers. Historically, HSV1 was found infrequently in genital HSV infections, but recent studies show an increasing prevalence up to 60%, possibly associated with increasing orogenital contact, highlighting the importance of HSV1 in human health [1], [2]. HSV infections may also lead to ocular herpes with symptoms varying from mild to severe stromal keratitis. Epithelial keratitis progresses to stromal keratitis in 25% of cases, which may result in loss of sight and blindness, and recurrent ocular HSV1 infections remain the major cause of viral induced blindness. HSV infection can also cause encephalitis, aseptic meningitis and atypical pneumonia [1], [3].

The prevalence of antibodies to HSV1 in the adult population ranges between 52 and 84% and reaches approximately 90% in European countries and in the United States respectively whereas respectively 4 to 24% and 22% of the European and American adult population is seropositive for HSV2 [4]–[6], with many patients suffering recurrent symptoms upon reactivation of the virus [7].

The current therapeutics against herpesviruses face a number of important drawbacks. The therapeutic benefit of acyclovir treatment for encephalitis is limited, where the mortality in newborns remains high at 15%, where only 29% of neonatal survivors show normal development following resolution of infection [8], [9]. An increasing problem is the occurrence of resistance to acyclovir and derivatives which threatens the effectiveness of the drugs and reduces the likelihood of favourable outcomes [10]–[12]. An alternative therapeutic option for treatment of stromal keratitis is hampered by a lack of knowledge concerning the mechanism by which the virus reaches the stroma through the basement membrane (BM) [7], [10]–[13]. These problems highlight an unmet medical need for improved or novel therapeutic options for HSV infection.

The respiratory portion of the nasal cavities is lined by a pseudostratified ciliated epithelium with goblet cells supported by the lamina propria, consisting of connective tissue with seromucous glands and a rich venous plexus. The lamina propria is continuous with the periosteum or perichondrium of bone or cartilage respectively, which form the wall of the nasal cavities. The BM is a layered structure of extracellular matrix which separates cells from the surrounding lamina propria and which provides a structural support for most epithelia. The structure is organised by glycoproteins and proteoglycans, of which the main components are laminins, type IV collagens, nidogens and heparan sulphate proteoglycans. The BM consists of two layers, the basal lamina (lamina densa) and reticular lamina (lamina reticularis or fibroreticularis). The lamina densa consists of laminin, type IV collagen, entactin and proteoglycans and is in direct contact with epithelial cell surfaces. The lamina reticularis consists of type III collagens, called reticular fibers, supports the lamina densa and forms the connection with the lamina propria [14]–[16].

The literature concerning HSV penetration of the mucosal BM is ambiguous at best, where most previous HSV-mucosal studies focussed on quantification of viral production in nasal swabs. Patel et al. [17]–[18] performed a histopathologic study of the depth of herpetic human skin lesions and observed that HSV causes lesions extending through the BM. However, no immunohistochemical detection of viral antigens was performed, preventing determination of whether the virus crossed the BM. Furthermore, it remains elusive whether the inflammatory infiltrate plays a role either in preventing virus spread or in damaging the surrounding tissue, including the BM. Herpes stromal keratitis has been reported both in humans and animals [19]–[20]. In mice, it has been stated that herpetic stromal keratitis appeared to be an immunopathogenic disease, rather than a result from direct viral replication; albeit with potential roles of viral factors. Nevertheless, the relationship epithelial-stromal disease remained unclear [20]. More recently, researchers from the same group demonstrated small amounts of HSV antigens in the stroma. How these antigens reached the stroma to cause stromal keratitis remained an open question [21]. Weeks et al. [22] defined the BM as a barrier to HSV and postulated that virus reaches the lamina propria via free nerve endings above the BM based on intradermal injections of HSV below the BM in mice, with no disease in 4 out of five animals. The authors also demonstrated that HSV1 was unable to pass through a reconstituted BM; results that are at variance with commonly observed HSV viremia during primary genital infection [23]. Furthermore, Berrington et al. [24] detected HSV DNA in peripheral blood, visceral organs and body cavities, albeit without viral culture studies to prove the presence of viable virus. However, samples were resistent to DNAase digestion, suggesting that the DNA was contained within virions. Also, to reach peripheral blood, virus must breach the BM. Additionally, several research groups demonstrated HSV particles in human corneal stroma [19]. However, whether the virus can actively penetrate the BM or relies on BM-damaging lesions to do so, remains unclear.

Whereas for animal herpesviruses, nasal mucosa is considered the primary site of replication, to our knowledge, the role of human nasal mucosa in primary HSV infection is unknown. First, it is impossible to study primary viral replication in humans during a challenge experiment. Secondly, studies of anatomical sites of HSV are to our knowledge restricted to one study on human cadavers at autopsy which identified the nasal mucosa as one reservoir of infection [25], implying a potential role for nasal mucosa in the herpesvirus life cycle.

In the present study, a human mucosa model consisting of nasal respiratory explants was characterised into detail. Successful explant models should retain viability, integrity and normal morphology for up to 4 days post cultivation to allow viral invasion experiments. Besides the general interest and potential of human explant models, the model can be used to study HSV1-nasal respiratory mucosa interactions. In this study, we address the controversial question of whether HSV1 is able to penetrate the BM. We determined the kinetics of HSV1 spread in human nasal respiratory mucosa in 3-dimensions and demonstrated that HSV1 penetrated the BM in human nasal respiratory mucosa, representing a significant novel insight into the pathogenesis of HSV1.

## Methods

### The human nasal respiratory mucosa explant model

#### Preparation, isolation and cultivation of human nasal respiratory explants

Human nasal mucosa from lower turbinate (concha nasalis inferior) was obtained at the time of surgical treatment of 3 persons with septal deviations and otherwise healthy nasal mucosa in the university hospital UZGent. All persons provided written informed consent and the ethics committee of the Ghent University Hospital approved the study.

Tissues were transported in phosphate buffered saline (PBS), supplemented with 1 mg/ml streptomycin (Certa), 1000 U/ml penicillin (Continental Pharma), 1 mg/ml kanamycin (Sigma) and 5 µg/ml fungizone (Bristol-Myers Squibb). Sections of mucosa of 16 mm^2^ were excised and placed with epithelial surface upwards on fine-meshed gauze for culture at an air-liquid interface at 37°C and 5% CO_2_, maintained with serum-free medium (50% RPMI (Gibco)/50% DMEM (Gibco) supplemented with 0.3 mg/ml glutamin (BDH Biochemical), 1 µg/ml gentamycin (Gibco), 0.1 mg/ml streptomycin (Certa) and 100 U/ml penicillin (Continental Pharma)). During the cultivation period, medium was not replaced.

Explants were collected for morphometric and viability analysis at 0, 24, 48, 72 and 96 h of cultivation of triplicate independent experiments.

### Analysis of viability - TUNEL assay

DNA fragmentation was detected using the In Situ Cell Death Detection Kit (TUNEL reaction) (Roche). Samples were analysed with a Leica DM RBE fluorescence microscope (Leica Microsystems GmbH). TUNEL-positive cells were counted in five randomly selected fields of 100 cells of both epithelium and lamina propria (Table 1).

**Table 1:** Occurrence of apoptosis in the epithelium and lamina propria in human nasal respiratory explants during in vitro cultivation.

	% TUNEL-positive cells at … h of in vitro cultivation				
	0	24	48	72	96
Epithelium	0.7±0.4	0.5±0.4	0.6±0.5	1.1±1.0	0.4±0.2
Lamina propria	2.1±1.8	1.1±1.3	2.5±1.3	2.3±1.4	1.4±0.9

#### Analysis of viability

Functional integrity: Ussing chamber technique and FITC dextran Functional integrity of the tissue was investigated before cultivation and at the end of the 96 h cultivation period at air-liquid interface on the gauze. To this end, explants were mounted in Ussing chambers at 0 h of in vitro cultivation (immediately after sampling) and after 96 h of in vitro cultivation and the potential difference (PD, mV) and transmucosal electrical resistance (R, Ω.cm^2^) were monitored. PD reflects the integrity of the cell membrane and the activity of the ion pumps. R reflects the integrity of the tight junctions. A decrease in resistance may indicate damage to the mucosa. Tight junctions restrict in a selective manner paracellular diffusion of ions and noncharged solutes. Therefore, in addition to the measurement of R to determine ion permeability, fluorescently labeled dextran, 4 kDa FITC dextran, was used to monitor permeability of noncharged solutes, to fully characterise the functional state of the tight junctions [26]–[27]. For the Ussing chamber set up, four Ag/AgCl electrodes were connected on one side to each chamber by 3 M KCl-agar bridges and on the other side to an external six-channel microcomputer-controlled voltage/current clamp. R was determined from voltage deflections in response to bipolar 50 µA current pulses generated for 200 ms and subsequently calculated from Ohm's law using the software package Clamp version 2.14 (Muβler Scientific Instruments). Data were corrected for the offset potential and resistance of the buffer solution (the cultivation medium), determined prior to the experiments. The fluorescently labelled dextran 1 mg/ml solution was added to the mucosal compartment at the beginning of the Ussing chamber experiment. Hundred µl samples were taken from the mucosal and submucosal compartment to determine the initial concentrations, at 0 h of the Ussing chamber experiment. Two h after adding the FITC dextran to the mucosal compartment, 100 µl samples were taken from both compartments to determine tight junction leakage. Fluorescence (optical density) was measured at each sampling point using a Fluoroskan Ascent FL fluorometer (Thermo Labsystems) with λ_exc_ 495 nm and λ_em_ 521 nm (Table 2).

**Table 2:** Fluorescence intensity of mucosal and submucosal compartment using FITC dextran as a molecular marker for tight junction damage.

		Fluorescence intensity	
		Mucosal compartment	Submucosal compartment
0 h in vitro cultivation	0 h after adding FITC dextran	595.3±25.7	0.5±0.1
	2 h after adding FITC dextran	559.1±31.9	0.5±0.0
96 h in vitro cultivation	0 h after adding FITC dextran	553.8±51.6	0.5±0.0
	2 h after adding FITC dextran	565.3±32.8	0.5±0.0

### Morphometric analysis - Light microscopy

Explants were fixed and stained as described previously [28].

**Figure 1:**
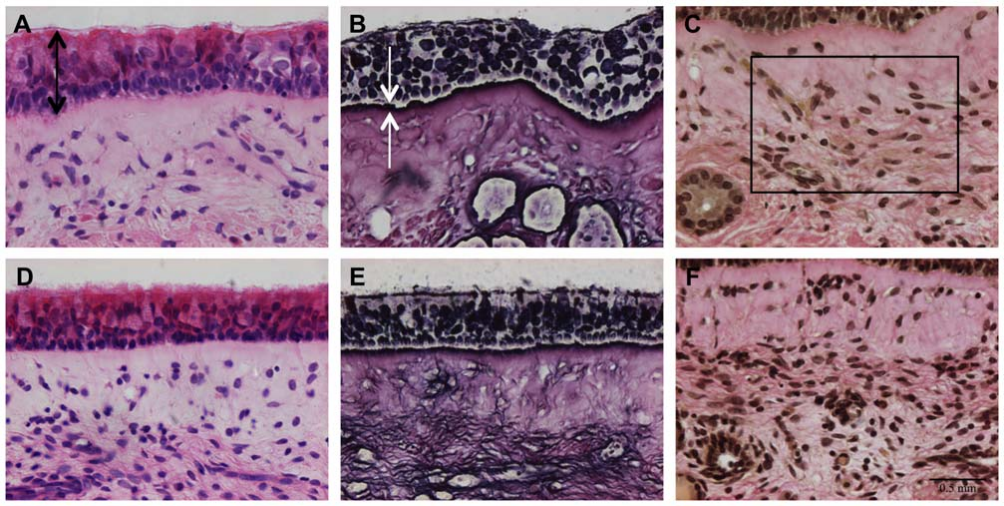
Light photomicrographs of the human model. Representative light photomicrographs of human nasal respiratory mucosa explants at 0 (A, B, C) and 96 h (D, E, F) of in vitro cultivation are illustrated. Light microscopical morphometric analysis was performed to evaluate the maintenance of structural integrity of the explants during in vitro cultivation. The three-dimensional organization of the explants was assessed by evaluating the morphometry of the epithelium, lamina reticularis and lamina propria. Eight-micron-sections were stained with haematoxylin-eosin (A and D) for evaluation of the epithelial thickness (indicated by black arrows). A reticulin staining (B and E) was performed to measure the thickness of the lamina reticularis (indicated by white arrows). A Van Gieson staining (C and F) was used to count the relative amounts of collagen and nuclei within a region of interest (roi indicated by a rectangle) of the lamina propria. By setting a threshold, different colors were assigned to collagen and nuclei, respectively, and the percentages of collagen and nuclei were determined within this roi. Bar, 50 µm.

**Figure 2:**
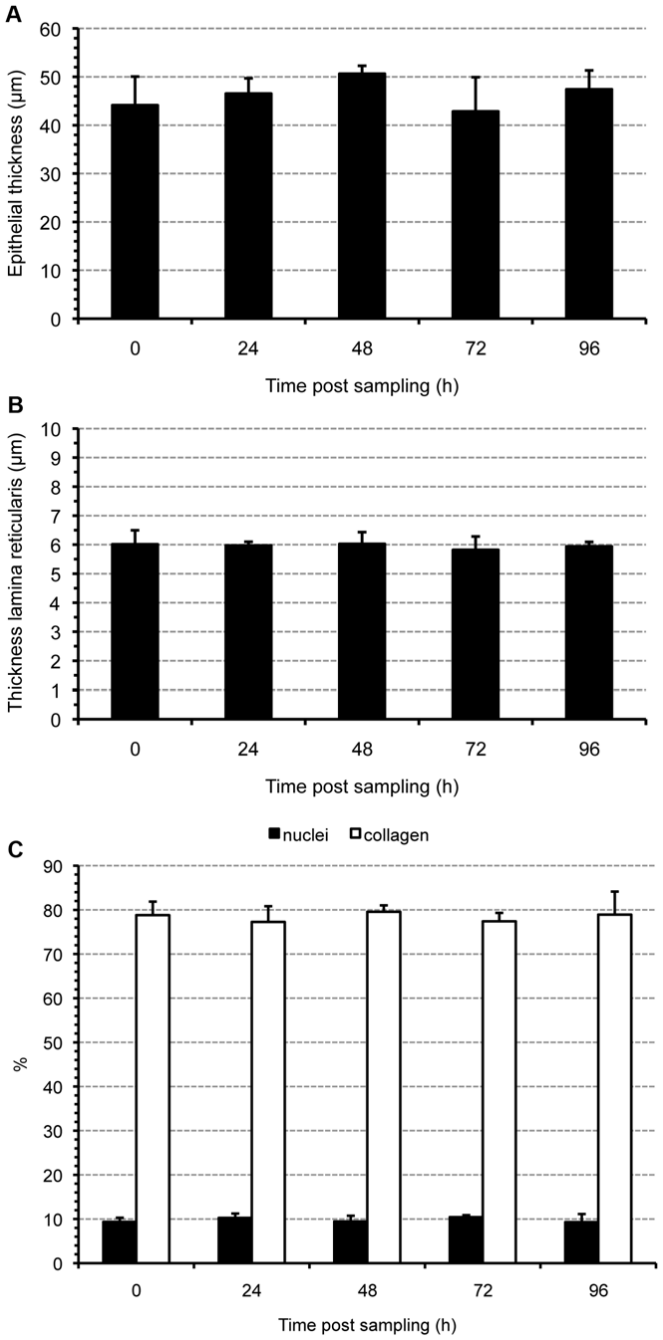
Morphometric evaluation of the human model. Maintenance of the structural integrity of the explants during in vitro cultivation was evaluated by assessing the three-dimensional organization of the explants. Epithelial thickness (A), thickness of the lamina reticularis (B) and percentages of nuclei and collagen within a region of interest of the lamina propria (C) were evaluated in explants at different time points of in vitro cultivation. Data are represented as means+SD (error bars) of triplicate independent experiments.

A haematoxylin-eosin staining was used to estimate the epithelial thickness at different time points of in vitro cultivation. As such, epithelial morphometry was evaluated for abnormalities (cell separation and extrusion, cell number, cell swelling). A reticulin staining was performed to visualise collagen type III reticular fibers, which are present in the lamina reticularis of the BM [29]. The reticular fibers (or reticulin) is a histological term used to describe a type of fibers in connective tissue composed of type III collagen [15], [30]. As such, type III collagen fibers or reticular fibers are not only part of the lamina reticularis of the BM, but also of the lamina propria. However, a higher concentration of type III collagen fibers forms a dense layer, which is defined as the lamina reticularis (marked with a white arrow in Figure 1). Using this stain, the thickness of the lamina reticularis was measured and as such, its continuity was evaluated during cultivation. The epithelial thickness and the thickness of the lamina reticularis were measured at five randomly selected places in five randomly chosen field. The Van Gieson staining marks all types of collagen, especially collagen type I [31] and was used to analyse the integrity of the lamina propria. Using the Soft Imaging System analySIS®, relative amounts of collagen and nuclei were calculated in a defined region of interest (roi) in five randomly selected fields by setting a threshold. All samples were analysed with an Olympus BX61 light microscope at magnification ×40 using the Soft Imaging System Cell^F^ (Olympus) (Figure 1 and Figure 2).

### Morphometric analysis - Transmission electron microscopy

**Figure 3:**
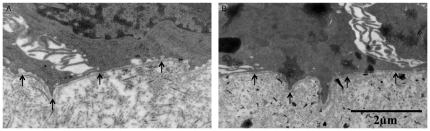
Transmission electron photomicrographs of the human model. The continuity of the lamina densa was evaluated during in vitro cultivation to assess the structural integrity of this basement membrane layer. The photomicrographs show representative images of the intact lamina densa in human nasal respiratory explants at 0 (A) and 96 (B) h of in vitro cultivation. Arrows indicate the lamina densa.

Fixation and embedding were performed as descibed previously [28]. Stained ultrathin sections were analysed using a JEM-1010 transmission electron microscope (Jeol Ltd.). The continuity of the lamina densa was visualised using transmission electron microscopy (Figure 3).

### Interactions between HSV1 and human nasal respiratory mucosa

#### Virus strain and inoculation procedure

HSV1 (ATCC VR-733) virus stocks were grown on Vero cells (ATCC CCL-81).

Explants were inoculated immediately after cultivation. Therefore, explants were placed at the bottom of a 24-well plate with epithelial surface upwards, washed twice with warm serum-free medium and inoculated by adding 1 ml virus suspension containing 10^7^ TCID_50_ HSV1/ml, which was obtained by diluting virus stock in serum-free explant cultivation medium to final concentrations of 10^7^ TCID_50_/ml (1 h, 37°C, 5% CO_2_). One h after inoculation, explants were washed three times and transferred again to the gauze. At 0, 12, 16, 20, 24 and 36 h post inoculation (pi), samples were collected, embedded in methocel® (Fluka) and frozen at −70°C. Explants of 3 different persons were included.

#### Evaluation of HSV1 mucosal spread - Immunofluorescence staining

Cryosections (20 µm) were made, fixed in methanol (−20°C, 100%, 25 min) and stained for HSV1 antigens and collagen IV to enable discrimination of the BM from the overlying epithelium. Cryosections were incubated (1 h, 37°C) with goat anti-collagen IV antibodies (Southern Biotech) (1∶50 in PBS), washed three times (PBS), incubated (1 h, 37°C) with biotinylated rabbit anti-goat IgG antibodies (Sigma) (1∶100 in PBS), washed three times (PBS) and incubated (1 h, 37°C) with streptavidin-Texas Red (Molecular Probes) (1∶50 in PBS). Subsequently, explants were incubated with 10% NGS (10 min, RT). HSV1-infected cells were detected by incubating (1 h, 37°C) the sections with mouse monoclonal anti-HSV1 gD antibodies (Santa Cruz Biotechnology) (1∶100 in 10% NGS), washing three times (PBS) and incubating (1 h, 37°C) with FITC-labelled goat anti-mouse antibodies (Molecular Probes) (1∶100 in 10% NGS). Finally, cryosections were washed three times (PBS) and mounted with glycerin-DABCO.

#### Evaluation of HSV1 mucosal spread - Confocal microscopy

Immunofluorescence image series of stained cryosections were acquired with a Leica TCS SP2 confocal microscope (Leica Microsystems GmbH). A Gre/Ne 543 nm and Argon 488 nm laser were used to excite respectively Texas Red- and FITC-fluorophores.

#### Evaluation of HSV1 mucosal spread - Plaque analysis

**Figure 4:**
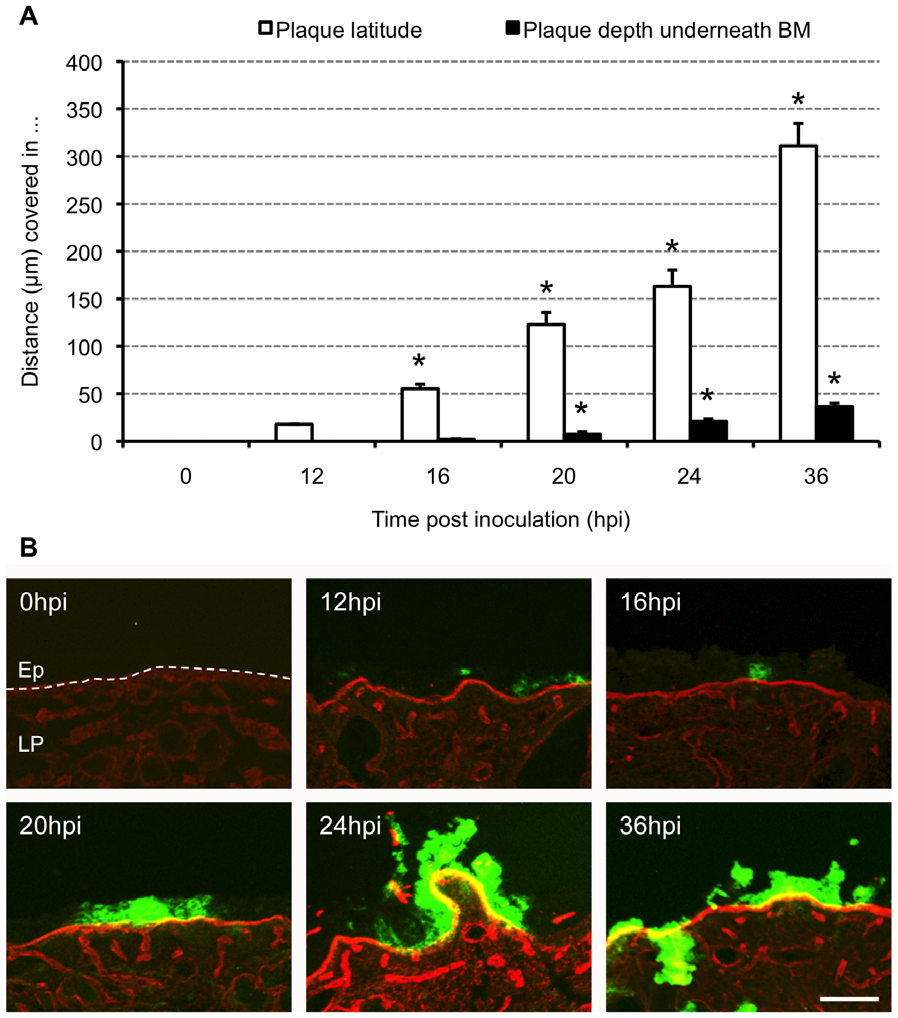
Evolution of HSV1 mucosal spread. (A) Kinetic evolution of HSV1 plaque formation. Explants were inoculated with 1 ml virus suspension containing 10^7^ TCID_50_/ml HSV1 VR-733 and sampled at 0, 12, 16, 20, 24 and 36 h post inoculation (pi). Serial 20 µm cryosections were made and plaque latitude (white bars) and plaque depth underneath the basement membrane (BM), distance covered by HSV1 in the lamina propria, (black bars) were measured using ImageJ. Data are represented as means of 10 plaques of triplicate independent experiments+SD (error bars). *, Significant differences at the 0.05 level. (B) Representative confocal photomicrographs of the evolution of HSV1 VR-733 spread in human nasal respiratory explants at 0, 12, 16, 20, 24 and 36 h pi. Collagen IV is visualised by red fluorescence. Green fluorescence visualises HSV1 antigens. Bar, 100 µm. Abbreviations: Ep, epithelium; LP, lamina propria. The BM is marked with a dashed line.

Plaque dimensions, latitude and penetration depth underneath the BM (distance covered by HSV1 in the lamina propria), were measured at maximal size for 10 different plaques per person at 0, 12, 16, 20, 24 and 36 h pi using the software imaging system ImageJ (Figure 4).

**Figure 5:**
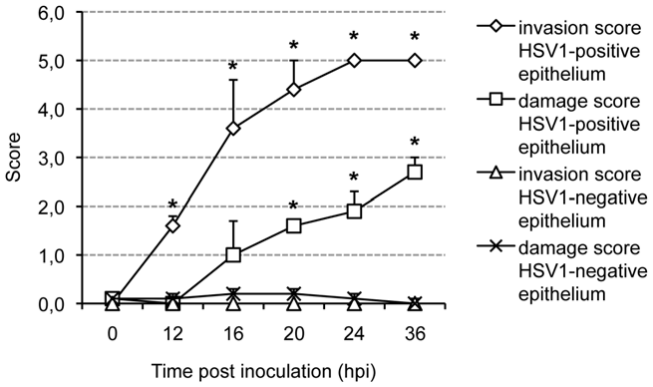
HSV1 invasion score and epithelial damage score. Explants were inoculated with 1 ml 10^7^ TCID_50_/ml HSV1 VR-733 and sampled at different time points post inoculation (pi). Serial 20 µm cryosections were made and evaluated. HSV1 mucosal invasion in the depth was graded on a 6-point scale, as follows: 0 = epithelium not infected, 1 = columnar cell(s) infected, 2 = suprabasal cell(s) infected, 3 = basal cell(s) infected, 4 = basement membrane and HSV1 colocalisation, HSV1 does not cross the basement membrane, 5 = HSV1 penetrates the basement membrane into the lamina propria. Epithelial damage was graded on a 4-point scale, as follows: 0 = no damage, 1 = superficial damage, 2 = epithelial damage involving basal cells, basal epithelial cells partly detached, 3 = epithelium severely damaged, loose. Both scales were combined on the same axis. The scores represent the mean of the scores of 10 different regions of HSV1-negative (100 cells) and HSV1-positive (virus plaque) cells per person at 0, 12, 16, 20, 24 and 36 h post inoculation (pi); experiments were performed in triplicate. Error bars indicate SD. *, Significant differences compared with the control (HSV1-negative epithelium) at the 0.05 level.

In addition, HSV1 mucosal invasion in the depth was graded on a 6-point scale (Figure 5). Analysis of the depth of HSV1 mucosal invasion (vertical spread perpendicular to the BM) was based on scoring the infected area, as follows: 0 = epithelium not infected, 1 = columnar cell(s) infected, 2 = suprabasal cell(s) infected, 3 = basal cell(s) infected, 4 = BM and HSV1 colocalisation, HSV1 does not cross the BM, 5 = HSV1 penetrates the BM into the lamina propria.

#### Epithelial damage in HSV1-infected human nasal respiratory mucosa

The epithelial damage of the immunofluorescence stained sections was assessed in 10 different populations of HSV1-negative and HSV1-positive cells, in extenso virus plaques at maximal size, per person at 0, 12, 16, 20, 24 and 36 h pi. Epithelial damage was graded on a 4-point scale (Figure 5).

#### Cell survival in HSV1-infected human nasal respiratory mucosa

For detection of HSV1 antigens, immunofluorescence stainings were performed as described above. The TUNEL assay (Roche) was performed to detect DNA fragmentation. The fluorescent nucleic acid dye, ethidium monoazide bromide (EMA, Invitrogen), selectively staining DNA in cells with a disrupted plasma membrane, was used at a concentration of 0.05 mg/ml as a marker for necrosis and late stages of apoptosis [32]–[33]. TUNEL- and EMA-positive cells were counted in regions containing HSV1-negative cells or HSV1-positive cells (virus plaque) at 0, 12, 24 and 36 h pi per person. For HSV1-negative cells, 10 randomly selected regions of 100 cells were evaluated for both epithelium and lamina propria. For HSV1-positive cells, 10 randomly selected HSV1 plaques were evaluated in both epithelium and lamina propria. Experiments were performed in triplicate.

### Statistical analysis

Data were statistically evaluated by SPSS software (SPSS Inc.) for analysis of variance (ANOVA). Results with P values of ≤0.05 were considered significant. Data shown represent means+SD of triplicate independent experiments.

## Results

### The human nasal respiratory mucosa explant model

#### Viability of epithelium and lamina propria

The effect of cultivation on the viability of the explants was verified by quantification of the percentage apoptotic cells in both epithelium and lamina propria at 0, 24, 48, 72 and 96 h of in vitro cultivation (Table 1). No significant differences were observed throughout cultivation. The percentage TUNEL-positive cells in epithelium and lamina propria ranged from 0.4 to 1.1% and from 1.1 to 2.5% respectively.

By using the Ussing chamber technique and FITC dextran, the maintenance of functional integrity of the explants during cultivation was verified. Mean changes ± SD in R and PD of triplicate independent experiments before and after 96 h of in vitro cultivation were determined by the Ussing chamber technique. We obtained a modest decline in R over time with a mean value of 377.7±12.5 Ω.cm^2^ before cultivation and a mean value of 315.3±12.7 Ω.cm^2^ after 96 h of in vitro cultivation. Mean PD values did not decrease with time of in vitro cultivation. We obtained a mean PD value of −1.2±3.1 mV and −0.2±0.6 mV before and after cultivation respectively. Using FITC dextran as a molecular marker of tight junction intact functionality, no time-dependent increase in the fluorescence intensity values of the submucosal compartment (Table 2) was measured after 96 h of in vitro cultivation.

#### Epithelial morphometry

No significant differences in epithelial thickness were observed over time. Representative images of epithelial thickness at 0 and 96 h of in vitro culture are illustrated in Figure 1a and 1d. Quantification is shown in Figure 2a.

#### Basement membrane morphometry

Two layers are present within the BM: lamina reticularis and lamina densa [34]. Therefore, morphometry of the BM was evaluated by measuring thickness of the lamina reticularis using light microscopy and by evaluating integrity of the lamina densa using transmission electron microscopy at specific time points post-sampling. No significant changes were observed in thickness of the lamina reticularis during the cultivation period (Figure 2b). Representative images of the lamina reticularis at 0 and 96 h post-sampling are illustrated in Figure 1b and 1e. The lamina densa remained continuous throughout cultivation (Figure 3).

#### Morphometry of the lamina propria

Integrity and composition of the lamina propria were evaluated using a Van Gieson staining (Figure 1c and 1f). No significant differences were observed in relative amounts of collagen and nuclei over time (Figure 2c).

### Interactions between HSV1 and human nasal respiratory mucosa

#### Evaluation of HSV1 mucosal spread

Inoculation of human nasal respiratory explants with HSV1 resulted in the occurrence of virus plaques. Kinetics of viral mucosal invasion were evaluated by measuring maximal plaque latitude and penetration depth underneath the BM at 0, 12, 16, 20, 24 and 36 h pi. Mean values+SD of three independent experiments are represented in Figure 4a. Plaque latitudes increased steadily over time, from 0.0 µm at 0 h pi to 17.7, 55.3, 122.9, 163.2 and 311.1 µm at 12, 16, 20, 24 and 36 h pi, respectively. Remarkably, HSV1 penetrated the BM rapidly where virus plaques crossed the BM from 16 h pi onwards and virus plaque size underneath the BM increased steadily from 0.0 µm at 0 h pi to 0.0, 1.8, 7.5, 20.8 and 36.2 µm at 12, 16, 20, 24 and 36 h pi, respectively. The depth of HSV1 mucosal invasion is illustrated in Figure 5. Representative confocal photomicrographs of plaque formation are illustrated in Figure 4b. About 20% of the viral plaques breached the BM at 16 h pi, increasing to 50% at 20 h pi and reaching 100% at 24 h pi demonstrating rapid penetration of the protective lamina by HSV1.

#### Epithelial damage in HSV1-infected human nasal respiratory mucosa

Comparisons of epithelial damage revealed aggravated epithelial damage and loosening of epithelial cells in HSV1-infected epithelium compared with baseline damage scores of regions of non-infected cells with increasing time pi (Figure 5 and illustrations Figure 4b).

#### Cell survival in HSV1-infected human nasal respiratory mucosa

After inoculation with HSV1, a large number of cells were HSV1-positive, where a minority of either HSV1-infected or non-infected cells were TUNEL- or EMA-positive (Table 3 and 4 respectively).

**Table 3:** Rate (%) of TUNEL-positive cells ± SD in both the epithelium and lamina propria of a region of HSV1-negative and of a region of HSV1-positive cells (virus plaque) at 0, 12, 24 and 36 h post inoculation (pi).

	% TUNEL-positive cells at … h pi			
	0	12	24	36
HSV1-negative epithelium	0.6±0.1	0.6±0.2	1.2±0.4	0.6±0.1
HSV1-negative lamina propria	1.0±0.1	0.9±0.4	1.0±0.3	1.2±0.6
HSV1-positive epithelium	ND	0.0±0.0*	1.0±0.8	3.1±1.6*
HSV1-positive lamina propria	ND	ND	8.9±7.4	5.6±3.9

ND = not determined; no HSV1-positive cells were found.

* Significant differences compared with the control (HSV1-negative tissue) at the 0.05 level.

**Table 4:** Rate (%) of EMA-positive cells ± SD in both the epithelium and lamina propria of a region of HSV1-negative and of a region of HSV1-positive cells (virus plaque) at 0, 12, 24 and 36 h post inoculation (pi).

	% EMA-positive cells at … h pi			
	0	12	24	36
HSV1-negative epithelium	0.4±0.1	0.3±0.2	0.4±0.2	0.3±0.2
HSV1-negative lamina propria	0.4±0.3	0.7±0.5	0.5±0.5	0.3±0.1
HSV1-positive epithelium	ND	0.8±1.4	1.2±0.3*	1.0±0.6
HSV1-positive lamina propria	ND	ND	0.1±0.2	0.5±0.5

ND = not determined; no HSV1-positive cells were found.

* Significant differences compared with the control (HSV1-negative tissue) at the 0.05 level.

## Discussion

Interactions of herpesviruses with mucosa remain poorly understood with a sparse knowledge of stromal invasion. Therefore, investigation of the mucosal invasion mechanism of alphaherpesviruses is pivotal in delivering new insights into the pathogenesis of these viruses, and ultimately, towards new therapeutic options.

So far, herpesvirus-mucosa interactions have been mainly studied in non-homologous animal models. In the current study, a homologous human explant model was used. In order to investigate mucosal invasion mechanisms of viruses, it is vital that the morphology and viability of the different tissue layers in the explant are maintained throughout the cultivation period. Explant models of human nasal mucosa have been described previously [35]–[38], where only Jackson et al. [37] performed a morphometric and viability analysis, albeit with no evaluation of the morphometry of the BM or viability and integrity of the lamina propria, both crucial factors when studying mucosal invasion through the BM. Where previous studies focused on epithelial cell viability and integrity, our study underscores the importance of an examination of the integrity and viability of both epithelium and lamina propria and of the examination of the continuity of the BM barrier.

A human nasal respiratory explant model was characterised analogous to a porcine nasal respiratory explant model that we reported recently [28]. Normal epithelial function depends upon the retention of cell junctions, the polarity of the epithelium and the orientation of the epithelium on the connective tissue substratum. Tissue function is maintained by normally constant architectural relationships. Loss of this three-dimensional organization can affect secretion and other physiologic responses [37]. The presence of BMs is a pre-requisite for most tissues to function properly as BMs have the capability to influence various activities of surrounding cells and may alter intercellular and cell-matrix junctions [14], [39]. Therefore, integrity and viability of both epithelium and lamina propria as well as BM integrity were evaluated. The structural integrity was evaluated by assessment of the three-dimensional organization. Thickness of epithelium and lamina reticularis, continuity of lamina densa and relative amounts of collagen and nuclei remained similar throughout the cultivation period. Viability and functional integrity of the explants were evaluated in both epithelium and lamina propria by quantifying DNA fragmentation (TUNEL assay) at 0, 24, 48, 72 and 96 h of in vitro cultivation, by quantifying plasma membrane permeability (EMA staining) at 0 and 36 h of cultivation, by performing the Ussing technique and using FITC dextran as a marker for tight junction leakage. In both epithelium and lamina propria, the percentage TUNEL or EMA-positive cells remained below 2.5 and 0.7% respectively. Using the Ussing chamber technique, we obtained a modest decline in R over time with a mean value of 377.7±12.5 Ω.cm^2^ before cultivation and a mean value of 315.3±12.7 Ω.cm^2^ after 96 h of in vitro cultivation. Mean PD values did not decrease with time of in vitro cultivation. We obtained a mean PD value of −1.2±3.1 mV and −0.2±0.6 mV before and after cultivation respectively. These values are largely in line with previously reported bioelectric properties of human nasal mucosal tissue or cultured human nasal epithelial cells. R values of 665±124 Ω.cm^2^ and 187±24 Ω.cm^2^ were acquired for human nasal epithelial cell monolayers obtained from healthy individuals undergoing corrective surgery of the nasal septum [40] and from individuals undergoing surgery for symptomatic nasal obstruction caused by nonatopic or allergic rhinosinusitis [41] respectively. PD values of −6±2 mV were shown for normal human nasal turbinate tissue obtained from individuals with healthy mucosa undergoing reconstructive surgery [42], whereas PD values for human nasal tissue obtained from individuals suffering from nasal obstruction resulting from nasal allergy reached −10 mV [26]. As PD values differ with the physiopathology of the nose, higher values were obtained for healthier mucosa, we can speculate that our initial PD values might correlate with healthy mucosa. More important is the fact that the PD value did not noticeably change over the cultivation period. Using FITC dextran as a molecular marker of tight junction intact functionality, no time-dependent damage could be demonstrated. Taken together, we can state that the functional integrity of the explants was largely retained during cultivation with a slight decrease in transmucosal electrical resistance.

The primary human nasal mucosa explants were susceptible to infection with HSV1. Morphometric characteristics of the HSV1 mucosal invasion process have not been determined previously. Using the explant model and a quantitative analysis system described previously [43], kinetics of HSV1 mucosal spread were determined by measuring evolution of plaque formation. HSV1 plaques were found to propagate in 3-dimensions and replication was observed in both epithelium and underlying lamina propria. HSV1 antigens were observed in the lamina propria from 16 h pi, where the time frame of BM crossing was between 12 and 24 h pi. All viral plaques had breached the BM at 24 h pi.

The viral crossing of the BM is consistent with observations made for the porcine alphaherpesvirus, pseudorabies virus (PRV), using the porcine model; viral spread through the BM evolved similarly with increasing time pi [43]. However, some differences were observed. Whereas PRV plaques show a distinct penetration of the BM encompassing the entire radius of the viral plaque; HSV plaques show a more limited, localized area of BM penetration, which may correlate with the more pronounced respiratory problems (mucopurulent nasal discharge) associated with PRV. Similar observations were made for bovine herpesvirus type 1 [44]. Viral crossing of the BM has not been reported for equine herpesvirus type 1, where plaques remain confined to the epithelium [45].

Studies on HSV1 mucosal invasion in human tissue are limited. Our observations for the first time unequivocally show that HSV1 has the capacity to breach the BM, where virus replication is found in the lamina propia underneath the BM. Notwithstanding the statement of Weeks et al. [22] that virus reaches the stroma via free nerve endings, we postulate that virus must traverse the BM to accomplish replication in the lamina propria, a position sustained by the fact that nerve fibres are surrounded by a BM barrier once they extend in the lamina propria [46]. The human nasal respiratory explant model may provide a pivotal role in exploring the mechanism of viral transport towards and through the BM. So far, it remains elusive whether or not the virus can directly manipulate host factors to facilitate taxis towards and penetration of the BM. The explant model represents a readily available, easily tractable and holistic tool to address these questions.

Effects of HSV1 infection on human nasal respiratory mucosa were evaluated using an epithelial damage scoring system and by performing a TUNEL assay and an EMA staining. It was shown that epithelial cells were loosened with increasing time pi. However, rounding up and detachment of HSV1-infected epithelial cells was not accompanied by apoptosis or necrosis. Only a few HSV1-positive cells were TUNEL- or EMA-positive, even at 36 h pi, although a large amount of the epithelial cells were HSV1-positive and showed the typical cytopathic effect. However, we have to remark that at later time points pi, some epithelial HSV1-infected cells were already lost. The amount of HSV1-positive cells in the lamina propria that were TUNEL-positive appeared to be higher, although not significantly, than in the epithelium. Our data are in line with Esaki et al. [47], who showed a large number of HSV-positive epithelial cells after corneal inoculation of mice, with only a minority being TUNEL-positive. In comparison, more TUNEL-positive cells were seen in the stroma. Asano et al. [48] revealed that HSV-positive epithelial cells remained TUNEL-negative after corneal infection of mice, though epithelial swelling was noticed and changes in apoptosis were observed in the connective tissue. Further along these lines, Miles et al. [49] obtained corneas from 2 patients with herpes simplex keratitis, where one infection was determined to be quiescent, the other one acute. Viral- and TUNEL-positivity was shown in epithelial cells in the acute infection and in stromal keratocytes in the quiescent infection, which might indicate that apoptosis does occur during HSV1 keratitis in humans. In this study, no percentage of TUNEL-positive cells relative to HSV1-infected cells was mentioned. Our data, derived during a primary HSV1 infection in our explant model system, demonstrated very low levels of apoptosis in HSV1-infected cells of both epithelium and lamina propria.

Previously, it has been demonstrated that herpesviruses effect a block in apoptosis during infection, playing a crucial role in viral pathogenesis [47], [50]–[51]. HSV1 encodes several proteins that counteract HSV infection-induced apoptosis. US3, US5, US11, ICP6 and the R1 subunit of HSV ribonucleotide reductase (LAT RNA) are able to counteract apoptosis in the absence of other viral functions [52]–[55]. Furthermore, ICP27 [56] and UL14 [57] are also involved in suppression of apoptosis. Studies describing the anti-apoptotic activity of these viral proteins were performed in cultured human cells and rabbits. Increasing evidence indicates that the effects, resulting from perturbations of the apoptotic process are cell type- and stimulus-specific [58]. In support of this, HSV1 was shown to block apoptosis in a cell-type-dependent manner [59]. Therefore, it is most interesting to obtain more in vivo data to understand the interaction between HSV and human tissues and to determine whether HSV1 is able to suppress apoptotic pathways in human tissues. Our current ex vivo data indicate that the mechanisms employed by HSV1 to counteract apoptosis are functional in nasal mucosal tissue.

Based on the limited number of TUNEL- or EMA-positive cells, we speculate that anti-apoptotic and/or anti-necrotic functions of HSV1 are active in nasal mucosa. Promoting epithelial cell survival would ensure that virus can penetrate the BM and reach the lamina propria before the epithelial cells become apoptotic and therefore, would enhance the depth of mucosal invasion in the infection process. The simultaneous rounding up of epithelial cells could facilitate virus release in the surroudings. Moreover, epithelial damage could play a role in promoting local bacterial infections.

In conclusion, an in vitro human nasal respiratory explant model was characterised, enabling research on HSV1 mucosal invasion. HSV1 was found to replicate both in epithelium and underlying lamina propria. Apoptotic or necrotic effects did not accompany the severe epithelial damage, which may underscore the anti-apoptotic and/or anti-necrotic features of HSV1. The evolution of HSV1 plaque formation was determined in 3-dimensions. Our results for the first time directly demonstrate that HSV1 penetrates the BM between 12 and 24 h pi. Future research will be aimed at unraveling the mechanisms of HSV mucosal invasion and BM passage. This research may lead to strategies, which interfere with early steps of herpesvirus pathogenesis by inhibiting stromal infection and therefore blocking mucosal invasion, which may complement current antiviral drugs for critical and sometimes life-threatening infections and offer alternative therapeutic approaches in the case of infections with resistant viruses.
